# Carbon Sequestration Efficiency of Organic Amendments in a Long-Term Experiment on a Vertisol in Huang-Huai-Hai Plain, China

**DOI:** 10.1371/journal.pone.0108594

**Published:** 2014-09-29

**Authors:** Keke Hua, Daozhong Wang, Xisheng Guo, Zibin Guo

**Affiliations:** Soil and Fertilizer Research Institute, Anhui Academy of Agricultural Sciences, Hefei, China; University of Maryland, United States of America

## Abstract

Soil organic carbon (SOC) sequestration is important for improving soil fertility of cropland and for the mitigation of greenhouse gas emissions to the atmosphere. The efficiency of SOC sequestration depends on the quantity and quality of the organic matter, soil type, and climate. Little is known about the SOC sequestration efficiency of organic amendments in Vertisols. Thus, we conducted the research based on 29 years (1982–2011) of long-term fertilization experiment with a no fertilizer control and five fertilization regimes: CK (control, no fertilizer), NPK (mineral NPK fertilizers alone), NPK+1/2W (mineral NPK fertilizers combined with half the amount of wheat straw), NPK+W (mineral NPK fertilizers combined with full the amount of wheat straw), NPK+PM (mineral NPK fertilizers combined with pig manure) and NPK+CM (mineral NPK fertilizers combined cattle manure). Total mean annual C inputs were 0.45, 1.55, 2.66, 3.71, 4.68 and 6.56 ton/ha/yr for CK, NPK, NPKW1/2, NPKW, NPKPM and NPKCM, respectively. Mean SOC sequestration rate was 0.20 ton/ha/yr in the NPK treatment, and 0.39, 0.50, 0.51 and 0.97 ton/ha/yr in the NPKW1/2, NPKW, NPKPM, and NPKCM treatments, respectively. A linear relationship was observed between annual C input and SOC sequestration rate (SOCsequestration rate  = 0.16 Cinput –0.10, R = 0.95, P<0.01), suggesting a C sequestration efficiency of 16%. The Vertisol required an annual C input of 0.63 ton/ha/yr to maintain the initial SOC level. Moreover, the C sequestration efficiencies of wheat straw, pig manure and cattle manure were 17%, 11% and 17%, respectively. The results indicate that the Vertisol has a large potential to sequester SOC with a high efficiency, and applying cattle manure or wheat straw is a recommendable SOC sequestration practice in Vertisols.

## Introduction

Soil organic carbon (SOC) sequestration contributes to the mitigation of greenhouse gas emissions and to the improvement of soil fertility [1]. Net SOC sequestration is the balance of organic C inputs into the soil (via crop residues, organic amendments in compost, animal manure, etc.) and organic C decomposition by soil microbes. SOC sequestration efficiency is commonly expressed by the relationship between annual C input and SOC accumulation rate, which is an indicator of soil C sequestration ability [2]. Therefore, information about the C sequestration efficiency is useful for seeking high efficiency management strategies of enhancing the SOC stock and soil fertility.

C sequestration efficiency is regulated by climate, the quantity, quality of added organic materials, and soil inherent properties [3]–[5]. Within a climate zone, soil inherent properties (i.e. initial SOC content, soil texture, clay type and aggregates) and cultivation practices have effects on SOC sequestration efficiency. A negative linear relationship has been reported between C sequestration efficiency and initial SOC content [6], [7], mainly because SOC tends to increase faster if initial SOC content is far from its saturation level. Feller and Beare [8] reported a positive relationship between SOC sequestration and soil clays. Because clay type (i.e. 2∶1 smectite clay minerals versus 1∶1 allophanic clay minerals) has influence on the stabilization of SOC [9]. Elliott and Coleman [10] indicated that soil aggregates may physically protect SOC against decomposition by soil microbes. Recently, several studies have examined the SOC sequestration efficiency of different soil types based on long-term fertilization experiment [11]–[15]. However, no information is available about SOC sequestration efficiency and C sequestration efficiencies of organic amendments in Vertisols [16], which occupy approximately 4.0 million km^2^ in Huang-Huai-Hai Plain of China. Vertisols are characterized by a low SOC content (less than 0.6% in the topsoil) a high clay content (more than 35%) and shrinking and swelling properties which contribute to self-mulching [17].

The purpose of this study was to evaluate the SOC sequestration efficiency under long-term fertilization practices in Vertisols, with a winter wheat–soybean double cropping system. Specific objectives were (1) to assess C input and SOC dynamics under various long-term fertilization practices (2) to estimate the SOC sequestration efficiency of different organic amendments.

## Materials and Methods

### Ethics Statement

The administration of the department of agricultural of Anhui Province gave permission for this research at the study site. We confirm that the field studies did not involve endangered or protected species.

### Site description

**Figure 1:**
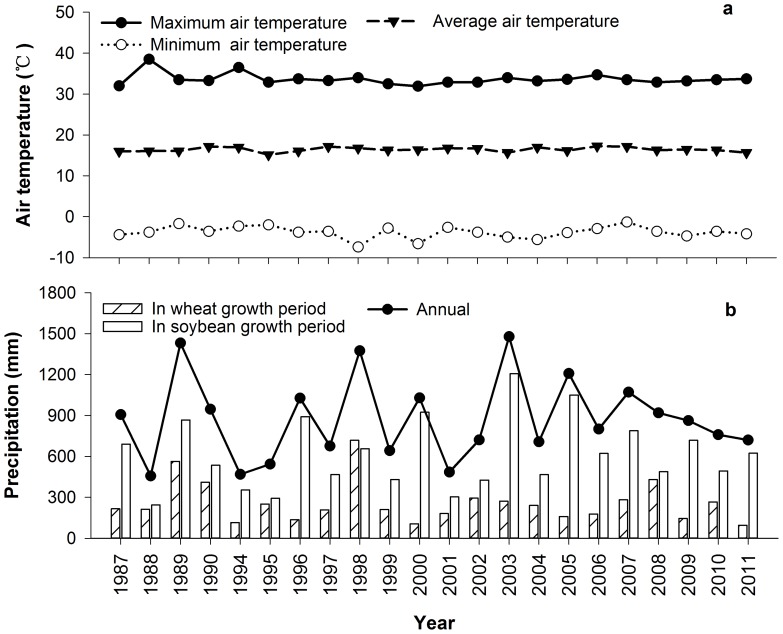
Air temperature and precipitation during the period 1987 to 2011 (no data for 1991 and 1993). (a) Maximum, minimum and average air temperatures. (b) Accumulated precipitation during the wheat growing period (from November to May), the soybean growing period (from June to October), and for the annual total.

The experiment is located at the Madian Agro-Ecological Station in Huang-Huai-Hai Plain, Eastern China (N33°13′, E116°37′). The areas has a sub-humid climate, with annual mean, maximum and minimum air temperature of 16.5°C, 36.5°C and −7.4°C, respectively (Fig. 1a). Annual precipitation ranged from about 457 to 1478 mm during the last 24 years (Fig. 1b), about 70% of which occurs from May to September. There are strong inter-annual and seasonal variations in precipitation. For example, a 90-day period of summer drought with a total precipitation of only 138 mm occurred in 1988. Precipitation in the soybean period ranged from 244 mm to 1049 mm with an average of 616 mm, which was much higher than that in the wheat period (259 mm).

The predominant Vertisols have developed in fluvial and lacustrine deposits. They are classified as Calcic Kastanozems, according to the soil classification system of the Food and Agriculture Organization (FAO). Soil pH ranges from 6.0 to 8.6 and SOC content from 5.8 to 7.5 g/kg in the area. The initial (1982) topsoil (0–20 cm) had a total SOC of 5.8±0.08 g/kg, total N 0.96±0.04 g/kg, total P 0.28±0.02 g/kg, bulk density 1.45 g/cm^3^ and pH (1∶2.5 w/v) 7.4, respectively. The topsoil under NPK treatment in 2013 had coarse sand (2 to 0.2 mm) 8.5±2.0 g/kg, fine sand (0.2 to 0.02 mm) 322.9±21.7 g/kg, silt (0.02 to 0.002 mm) 262.3±24.0 g/kg and clay (<0.002 mm) 406.3±24.0 g/kg, respectively.

### Experimental design

The long-term field experiment was initiated in 1982. The experiment had six treatments (Table 1): no fertilizer (CK), mineral nitrogen-phosphorus-potassium fertilizers alone (NPK), mineral NPK fertilizers combined with 2.5 ton/ha/yr (dry base) of wheat straw (NPKW1/2), mineral NPK fertilizers combined with 5.0 ton/ha/yr of wheat straw (NPKW), mineral NPK fertilizers combined with 7.8 ton/ha/yr of pig manure (NPKPM), and mineral NPK fertilizers combined with 12.5 ton/ha/yr of cattle manure (NPKCM). The treatments were laid out in a randomized block design with four replications. The net plot size was 70 m^2^ (14.9 m ×4.7 m). Each plot was isolated by 50 cm deep plates. Mineral N, P and K fertilization was urea, calcium superphosphate and potassium chloride, respectively. The amounts applied were similar to the amounts applied by local farmers, i.e., 180 kg N, 90 kg P_2_O_5_, and 135 kg K_2_O/ha/yr. All fertilizers were applied as base fertilizer at the start of the wheat growing season. No fertilizers were applied to soybean growing season because of the high background value of soil N, P and K, which could supply sufficient available nutrients for the growth of soybean. Moreover, extra nitrogen was also obtained by nitrogen fixation by soybean itself. The mean chemical characteristics of pig manure were 360 g C/kg (dry base), 17 g N/kg and 8.9 g P/kg, and those of cattle manure were 370 g C/kg, 7.9 g N/kg and 4.2 g P/kg.

**Table 1:** Mean application rates of mineral and organic fertilizers (kg/ha/yr) per treatment.

Treatment	N	P_2_O_5_	K_2_O	Wheat straw	Pig manure	Cattle manure
CK	0	0	0	0	0	0
NPK	180	90	135	0	0	0
NPKW1/2	180	90	135	2500	0	0
NPKW	180	90	135	5000	0	0
NPKPM	180	90	135	0	7800	0
NPKCM	180	90	135	0	0	12500

Treatments were no fertilization (CK), mineral NPK fertilizers alone (NPK), mineral NPK fertilizers combined with 2.5 ton/ha/yr of wheat straw (NPKW1/2), mineral NPK fertilizers combined with 5.0 ton/ha/yr of wheat straw (NPKW), mineral NPK fertilizers combined with pig manure (NPKPM), and mineral NPK fertilizers combined with cattle manure (NPKCM).

A double cropping system of winter wheat-soybean is the common crop rotation in the region. At the Madian Agro-Ecological Station, plots were plowed to 20 cm depth after crop harvest. Winter wheat (*Triticum aestivum L*.), variety Yedan 13, was grown in rows from late October to May, and soybean (*Glycine max*), variety Zhonghuang 13, from June to September. Herbicides and pesticides were applied during the growth periods when it was needed. Weed residues in all the fields were manually removed after herbicides application. Hence, biomass of weeds left in the field was negligible. Wheat and soybean were harvested manually and all above-ground biomass, except for stubble, were removed from the experimental fields. Wheat grain yields were measured in 1983, 1985, and 1989–2011. Soybean grain yields were measured in 1983, 1985, 1989–1991 and 1998–2011. Grains were air dried, threshed and then weighted separately.

### Soil sampling and analyses

Soil samples were collected from the top 20 cm each year after the soybean harvest (in October). Soil samples were randomly taken from three locations in each plot using a soil core sampler (inner diameter 7 cm). The samples were air dried and passed through an 8 mm sieve. Visible pieces of crop residues and roots were removed. Representative sub-samples were then passed through a 2 mm sieve and ground further to pass a 0.25 mm sieve for determination of SOC, total N, total P and other properties. The sieved soil was stored in glass jars until analysis. Soil bulk density was measured using the core method [18]. Soil pH was measured by the potentiometric method in a soil-water extract (1∶2.5, w/v water) [18]. SOC content was determined following the vitriol acid-potassium dichromate oxidation method [19]. Total N was determined by using the method described by [19], and total P by Black [20]. Soil texture was measured following the gravitometer methods described by Lu [18]. Four replicates were carried out for each analysis.

### Estimation of C inputs, and SOC sequestration rate and efficiency

C inputs into the topsoil (0–20 cm) included materials from crops residues (i.e., mainly roots and stubble), organic amendments (i.e. wheat straw, pig manure, and cattle manure). Annual amount of C inputs by roots from wheat and soybean were estimated at 30% [21] and 19% [22] of above-ground biomass, respectively. Annual C inputs by stubble from wheat and soybean were estimated at 15% of straw biomass. We used a straw to grain ratio of 1.2∶1 and 1.6∶1 to calculate wheat and soybean straw biomass, respectively [23]. The C contents of wheat and soybean were estimated at 400 g/kg and 453 g/kg (dry base), respectively [23]. The annual C input via crop residues was estimated from
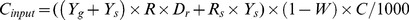
(1)where *Y*_g_, and *Y*_s_ were grain yield and straw yield (kg/ha); *R* was ratio of root biomass to above ground biomass (AGB); *D*_γ_ was ratio of root biomass in topsoil (0–20 cm) to root biomass in the soil profile, which were set at 0.753 for wheat [24] and at 0.984 for soybean [25]; *R*_s_ and *W* represented ratio of stubble to straw biomass and water content of air-dried grain (14% [23]); *C* was the organic C content of the crop (g/kg).

SOC stock (*SSOC*, ton/ha) in topsoil was calculated using the equation:
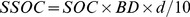
(2)where *SOC* was soil organic C content (g/kg), *BD* was the bulk density (g/cm^3^), and *d* was the thickness of the soil layer (20 cm).

SOC sequestration rate (*SSR*, ton/ha/yr) was estimated for the topsoil by the following equation [4]:
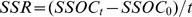
(3)where *SSOC_t_* and *SSOC_0_* were the stock of SOC at time *t* and in the initial year (1982); *t* was the duration of experiment. The SOC sequestration efficiency was derived from the slope of the linear regression between annual SOC sequestered and C input [14].

ΔSOC sequestration rates (Δ*SSR*, ton/ha/yr) via wheat straw, pig manure and cattle manure were estimated for topsoil by the following equation.
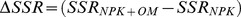
(4)where *SSR_NPK+OM_* and *SSR _NPK_* were the SOC sequestration rate in treatments with organic amendments combined with mineral NPK fertilizers (NPKW1/2, NPKW, NPKPM and NPKCM) and the treatment with mineral NPK fertilizers alone (NPK), respectively.

C sequestration efficiency of organic amendment (SEO) was calculated by the following equation.
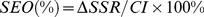
(5)where *CI* was annual C input via organic amendment (ton/ha/yr).

### Data analysis

All the statistical analyses were performed using SPSS 13.0 software package (SPSS, Inc., USA). Significant differences were analyzed using *LSD* test at significance level *P* = 0.05 or *P* = 0.01. Graphs were prepared with Sigma plot 10.0 software (Systat Software, Inc., Chicago, IL, USA).

## Results

### Grain yield and C inputs into the soil

**Figure 2:**
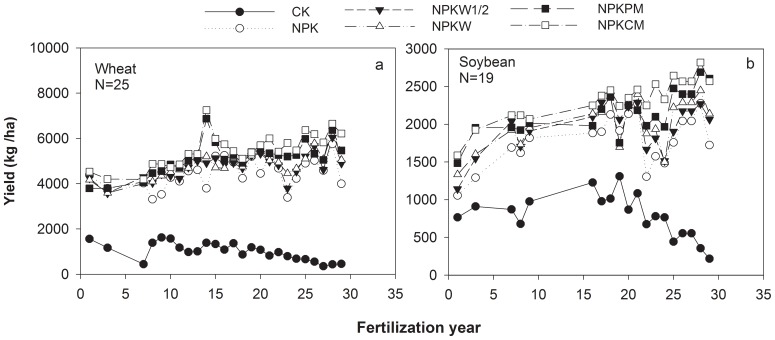
Average annual grain yields in six treatments during the period 1983 to 2011. (a) Wheat yields (b) Soybean yields. Treatments were no fertilization (CK), mineral NPK fertilizers alone (NPK), mineral NPK fertilizers combined with 2.5 ton/ha/yr of wheat straw (NPKW1/2), mineral NPK fertilizers combined with 5.0 ton/ha/yr of wheat straw (NPKW), mineral NPK fertilizers combined with 7.8 ton/ha/yr of pig manure (NPKPM), and mineral NPK fertilizers combined with 12.5 ton/ha/yr of cattle manure (NPKCM). Results are means (n = 4).

Wheat and soybean yields are shown in Fig. 2. Average annual grain yields followed the order NPKCM> NPKPM> NPKW> NPKW1/2> NPK> CK. Grain yields tended to decrease under the CK treatment, but to increase under all fertilization treatments over time.

Similarly, the CK treatment had a low and decreasing crop C input, while the fertilization treatments had a high and increasing trend of C input via roots and stubble over time (Table 2). Average annual C input via roots and stubble was 2.4 times higher in the NPK treatment than in the CK treatment. Soil amendments with straw and manure slightly increased the C inputs via roots and stubble in the NPKW1/2, NPKW, NPKPM and NPKCW treatments, respectively, comparing to the NPK treatment. Similarly, CK had the lowest annual total C input t (0.45 ton/ha). Compared with CK, the average annual total C inputs in the NPK, NPKW1/2, NPKW, NPKPM and NPKCW treatments were increased by a factor of 2.4, 4.9, 7.2, 9.4, and 13.6, respectively.

**Table 2:** Average annual crop C input (roots and stubble) and organic amendment C inputs for each treatment during the period 1987 to 2011.

Treatment	Annual crop C input (ton/ha/yr)				Organic amendment C input	Total
	Wheat	CV%	Soybean	CV%	(ton/ha/yr)	(ton/ha/yr)
CK	0.23c	38.3	0.22c	36.2	0	0.45
NPK	1.04b	14.3	0.51b	19.0	0	1.55
NPKW1/2	1.10b	11.8	0.56b	16.7	1.00	2.66
NPKW	1.13b	12.5	0.57b	16.1	2.00	3.71
NPKPM	1.20a	13.7	0.61a	14.0	2.87	4.68
NPKCM	1.28a	13.5	0.66a	12.3	4.62	6.56

Treatments were no fertilization (CK), mineral NPK fertilizers alone (NPK), mineral NPK fertilizers combined with 2.5 ton/ha/yr of wheat straw (NPKW1/2), mineral NPK fertilizers combined with 5.0 ton/ha/yr of wheat straw (NPKW), mineral NPK fertilizers combined with 7.8 ton/ha/yr of pig manure (NPKPM), and mineral NPK fertilizers combined with 12.5 ton/ha/yr of cattle manure (NPKCM). Data are present as means (n = 4). Those with the same letter are not significantly different (*P*<0.05 or 0.01).

### SOC dynamics

**Figure 3:**
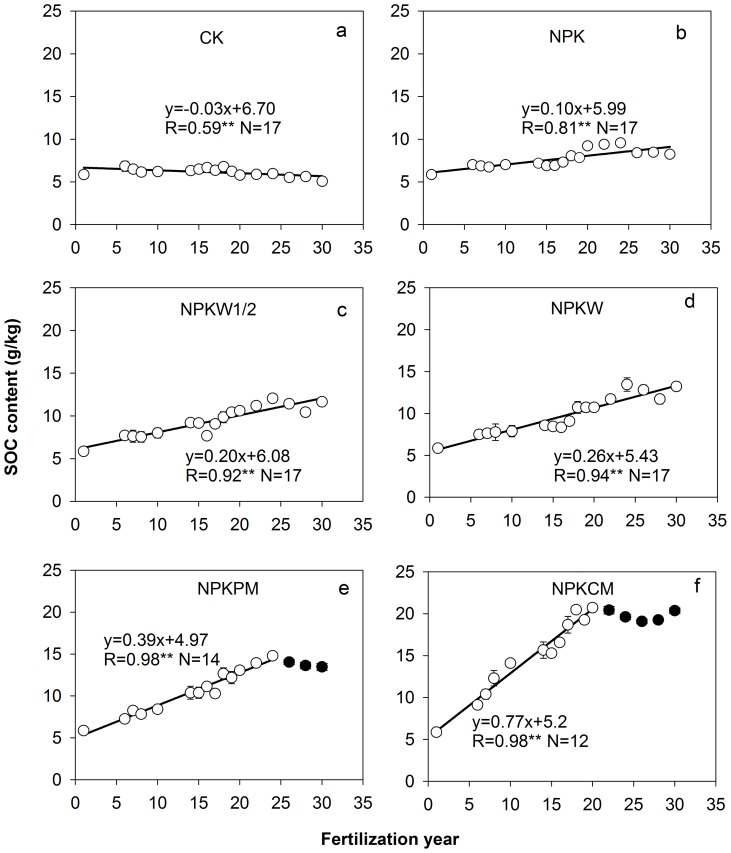
Changes in soil organic carbon (SOC) content over time in the six fertilization treatments during the period 1983 to 2011. CK: no fertilizer (a); NPK: mineral NPK fertilizers alone (b); NPKW1/2: mineral NPK fertilizers combined with 2.5 ton/ha/yr of wheat straw (c); NPKW: mineral NPK fertilizers combined with 5.0 ton/ha/yr of wheat straw (d); NPKPM: mineral NPK fertilizers combined with pig manure (e); NPKCM: mineral NPK fertilizers combined with cattle manure (f); ** indicates significant correlation at *P*<0.01, respectively. Error bars indicated for some years are standard errors (n = 4).

Changes in SOC contents are shown in Fig. 3. As expected, CK had a low and slightly decreasing SOC content, whereas other treatments had an increasing SOC content. Changer over time in SOC content could be described by linear regression or linear plateau models. In CK, the changes in SOC content were best described by simple linear regression (*y* =  −0.03 *x*+6.70, *R* = 0.59, *P<*0.01), suggesting that SOC decreased by 3% per year. Changes in SOC contents of the NPK, NPKW1/2 and NPKW were also best described by simple linear regression, but here significant increases in SOC content were observed. Changes in SOC contents of the NPKPM and NPKCM were best described by linear plateau model. For NPKPM and NPKCW, SOC contents tended to increase rapidly in the first twenty years, but leveled off thereafter. However, a longer duration of soil C sequestration was observed in NPKPM (26 years) than in NPKCM (20 years).

### C sequestration rate

Soil bulk density (BD) decreased in all fertilization treatments but not in the CK treatment (Table 3). The decrease was largest in the treatments receiving wheat straw and animal manures, which suggests that long-term application of organic amendments significantly improved soil physical conditions.

**Table 3:** Soil bulk density (BD), SOC contents, the estimated mean SOC sequestration rate (SSR) and mean ΔSOC sequestration rate (ΔSSR) during the 29-years period, for all treatments.

Treatment	1982			2011			SSR (ton/ha/yr)	ΔSSR (ton/ha/yr)
	BD (g/cm^3^)	SOC (g/kg)	SSOC (ton/ha)	BD (g/cm^3^)	SOC (g/kg)	SSOC (t/ha)		
CK	1.45	5.86	16.99	1.44±0.03a	5.08±0.17e	14.63±0.79e	−0.08±0.03e	-
NPK	1.45	5.86	16.99	1.38±0.01a	8.24±0.07d	22.74±0.47d	0.20±0.02d	-
NPKW1/2	1.45	5.86	16.99	1.21±0.02b	11.66±0.46c	28.22±1.58c	0.39±0.05c	0.19±0.03
NPKW	1.45	5.86	16.99	1.19±0.02b	13.23±0.55b	31.49±1.84b	0.50±0.06b	0.30±0.04
NPKPM	1.45	5.86	16.99	1.18±0.02b	13.46±0.42b	31.77±1.53b	0.51±0.05b	0.31±0.03
NPKCM	1.45	5.86	16.99	1.11±0.01c	20.36±0.32a	45.20±1.12a	0.97±0.04a	0.77±0.02

CK: no fertilizer; NPK: mineral fertilization; NPKW1/2: mineral fertilizer combined with 2.5 ton/ha/yr of wheat straw; NPKW: mineral fertilizer combined with 5.0 ton/ha/yr of wheat straw; NPKPM: mineral fertilizer combined with pig manure; NPKCM: mineral fertilizer combined with cattle manure. Data are presented as means (n = 4), and those designated with the same letter are not significantly different (*P*<0.01).

The mean SOC sequestration rate over the 29 years experimental period ranged from −0.08±0.03 ton/ha/yr in CK to 0.97±0.04 ton/ha/yr in NPKCM. Mineral fertilizer application (NPK) reversed the SOC decline in the CK treatment into a net SOC sequestration of 0.20±0.02 ton/ha/yr. Mean SOC sequestration rates followed the order: NPKCM> NPKPM> NPKW> NPKW1/2> NPK. These findings are consistent with a large body of evidence indicating that long-term fertilizer, crop residues and manure applications increase the SOC content of arable soils [4], [14]. Average ΔSOC sequestration rates (ΔSSR) of wheat straw at low input rate, wheat straw at high input rate, pig manure and cattle manure were 0.19±0.03, 0.30±0.04, 0.31±0.03 and 0.77±0.02 ton/ha, respectively. The result indicated that SOC sequestration rate changes were related to total C inputs.

### Relationship between C input and SOC sequestration rate

**Figure 4:**
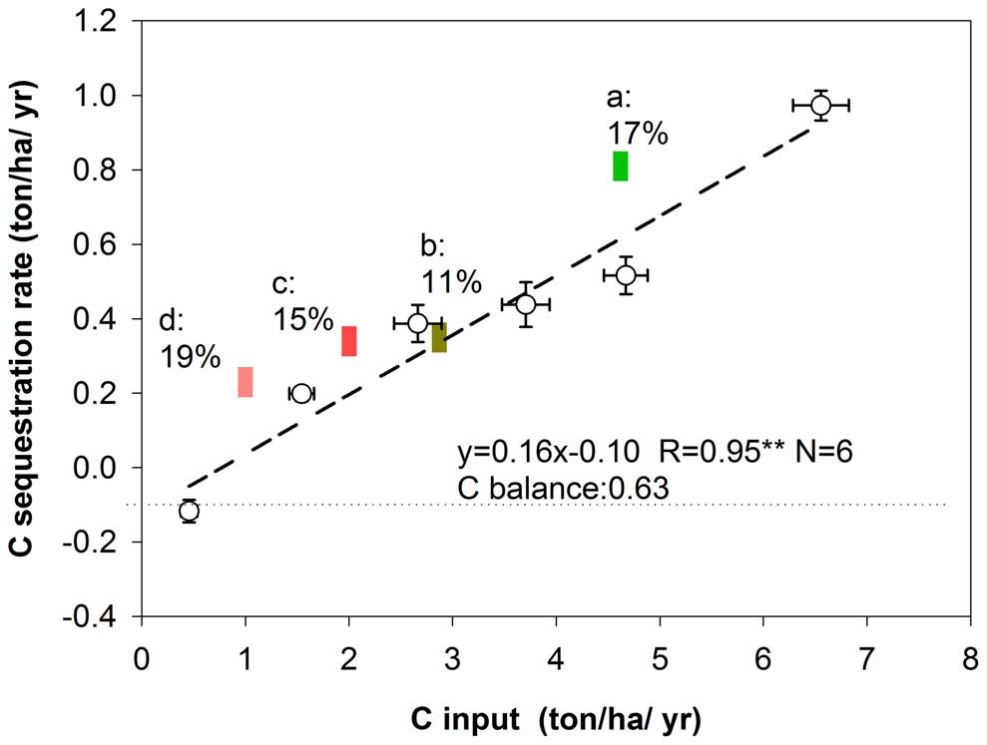
Relationship between C input and SOC sequestration rate. Solid line indicated linear regression curve between C inputs and SOC sequestration rates for the six treatments. a, b, c, and d represent C sequestration efficiencies for wheat straw at low input rate, wheat straw at high input rate, pig manure and cattle manure (calculated by equation (5)), respectively. ** indicated significant at *P*<0.01; error bars indicate standard errors (n = 4).

There was a significantly positive linear correlation between C input and SOC sequestration rate (Fig. 4). The slope of the equation (0.16) indicates that on average 16% of the total C input into the soil was sequestered as SOC; the mean soil C sequestration efficiency was 16%. The results also indicate that 0.63 ton/ha/yr was needed to maintain SOC level constant at the initial level.

The mean relationships between C inputs and **Δ**SOC sequestration rates through organic amendments are also shown in Fig. 4. Interestingly, C sequestration efficiencies for wheat straw (c and d), pig manure (b) and cattle manure (a) differed. The C sequestration efficiencies for wheat straw was 17% (mean value), pig manure 11% and for cattle manure 17%. Hence, application cattle manure or wheat straw has a higher efficiency to enhance SOC.

## Discussion

### Soil C sequestration duration

Our results clearly show that trends in SOC depend on long-term fertilization regime. The changes of SOC content over time for the three treatments of NPK, NPK1/2W and NPKW are consistent with the observations reported by Zhou et al (2013) [26]. The time of soil C steady state occurring represents soil C sequestration duration [27]. West and Six [28] reported that the duration of C sequestration varies between ecosystems, climate regimes, and fertilization management (e.g., soil organic amendments inputs). West and Post [29] estimated that a change from conventional tillage to no-till results in an average 14% increase in soil C, during a mean experimental period of 15 years. Rui and Zhang [30] found that there was a negative correlation between soil C sequestration rates and duration of soil C sequestration. The C sequestration duration in the NPKW, NPKPM and NPKCM treatments reflect differences in the quantity and quality of C input. Our results indicate that the recommended mean C sequestration period of 20 years for National Greenhouse Gas according to the IPCC Guidelines [31] may not be suitable for all the organic amendments application.

### Relation between C input and soil C sequestration

The relationship between C input into the soil and SOC sequestration may present in the SOC sequestration efficiency. Several studies showed that there was no general relationship between C input and SOC sequestration. Some soils might be C saturated [32]–[34]. Other studies demonstrated that SOC sequestration was linear or logarithmically related to the C input into soil [2], [4], [15]. In the present study, a clear linear relationship between annual C input and SOC sequestration rate was found, which was similar to findings in upland [4], [35], but different from those findings in paddy soils [14], [15], [36]. The strong linear relationship indicated that the Vertisol had a large capacity to store SOC. Moreover, we did not find indications that the Vertisol had a clear, uniform saturation level for SOC sequestration. The results presented in Fig. 3b, c and d indicate that these maxima (equilibrium levels) have not been reached in 29 years. However, we observed that SOC sequestration rate leveled off after about 20 years in the NPKPM and NPKCM treatments at SOC levels of about 15 and 20 g/kg. The results suggest that SOC saturation level depends on C inputs of organic amendments.

Our results revealed that 0.63 ton/ha/yr (Fig. 4) was needed to maintain the initial SOC level, and that the unfertilized control treatment (CK) did not provide sufficient C input to maintain the initial SOC content. This finding is in agreement with the result from Yan et al. [15], who showed that the C input from unfertilized crops cannot sustain the native SOC content of upland soils. Interestingly, NPK fertilization did provide sufficient C input to contribute to a net SOC sequestration, indicating that the C input provided through the incorporation of only roots and stubble was sufficient to maintain and even enhance the C stock. This contrasts with result reported by Yang [37]. They reported that NPK fertilization was insufficient for maintaining the SOC level under conditions of traditional management with the removal of all aboveground crop biomass. These differences may relate to differences in crop yield levels and in SOC sequestration efficiency. Our study indicates C inputs from wheat and soybean stubbles and roots under conventional NPK fertilization seem sufficient to replenish the C loss through SOC decomposition in Vertisols.

### C sequestration efficiency

The initial SOC content and soil texture (i.e. clay content) are important factors regulating SOC sequestration efficiency [9]. The Vertisol in our study had a low initial C content and a high clay content, which suggest that the SOC sequestration might be high. Indeed, we observed a relatively high efficiency irrespective of the type of C input (Fig. 4). Zhang [14] reported a C sequestration efficiency of 6.8% for alluvial soils in Zhengzhou, China, in the same climatic zone as our study. Stewart [38] argued that a greater SOC saturation deficit results in greater sequestration efficiency of added C. We estimated the soil C saturation deficit of the Vertisol at 80%, using the relationship between soil mineral (silt + clay) and C contents (g/kg soil) as proposed by Stewart [33]:
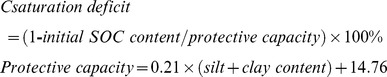



Evidently, the high clay+silt content and the low initial SOC content contributed to the relatively high C saturation deficit. The protection of SOC by clay particles was well established [39], [40]. Stewart [38] reported a larger enrichment of microbial derived carbohydrates in the clay fraction compared with that of the sand fraction. Hassink [41] reported that C associated with organo-mineral complexes in soil were chemically protected, and the protection increased with an increase in clay content. Six [9] found that soils with a high clay content had a low SOC decomposition rate, likely because SOC in clay soils was chemically stabilized and absorbed onto negatively charged clay minerals [42], [43]. Therefore, the clay content has a positive effect on SOC sequestration efficiency [44].

Many factors have been suggested to affect the humification coefficient, including the type of input material, soil type, climate factors and soil nutrients status [45], [46]. Maillard and Angers [47] reported that the quality of the organic amendment was the dominant driver determining C sequestration efficiencies of organic amendments, based on a meta-analysis of worldwide published reports. Likely, the quality of the organic amendment in our experiment was also the major reason explaining the differences in C sequestration efficiencies for wheat straw, pig manure and cattle manure. Especially lignin is considered to be one of the most chemical recalcitrant components [48]. In soils, lignins are synthesized from L-phenylalanine and cinnamic acids via various metabolic ways to form lignin precursors such as sinapyl and coniferylalcohols [49]. The lignin structure consists of aromatic rings with side chains and –OH and –OCH_3_ groups linked by strong covalent bonds. Therefore, lignins are considered as stabilized component of SOC, influencing its pool-size and its turnover [50]. In our study, we observed that C sequestration efficiency of cattle manure was higher than that of pig manure, likely because the cattle manure was composted. Ghosh et al. [51] reported that the lignin content of cattle manure may reach 23.7%, which was significantly higher than that of pig manure. Therefore, application of composted cattle manure seems the preferred strategy for enhancing SOC sequestration in the Vertisol due to its high C sequestration efficiency.

There are a number of uncertainties in our study, which are quite common to many long-term field studies. We estimated the C inputs via roots and stubble from fixed percentages of aboveground biomass. Likely, the C input via roots and stubble depends on the level of fertilization, and varies from year to year due to variations in rainfall. Also, the C content of the pig and cattle manure was not measured each year, while there was quite a big annual variation in composition. Further, there are missing data, especially during the first half of the experiment, due to organizational matters. We obtained C sequestration efficiencies of wheat straw, pig manure and cattle manure based on the two main hypotheses. We assumed that the difference in C input via stubble and roots was negligible small between the treatment NPK and the treatments NPKW1/2, NPKW, NPKPM and NPKCM, and no significant priming effect caused by organic amendments application was existed. These uncertainties in primary data may also lead to uncertainties in the estimated mean SOC sequestration efficiency and C sequestration efficiencies of organic amendments, but do not undermine our main conclusions. Our future studies focus on the accurate quantification of C inputs via roots and stubble, effects of organic amendments application on the C inputs, and the accurate quantification of priming effect by organic amendments application using isotope technique in this ongoing field experiment. Also, SOC fractionation studies are undertaken to establish relationships between C input and SOC sequestration in different SOC pools (i.e. light and heavy C fractions).

### Conclusions

The changes in SOC content over time were described by linear equations. However, the SOC sequestration rates clearly decreased in the NPKPM and NPKCM treatments after about 20 years. A significant linear relationship was observed between annual C input and SOC sequestration rate. The Vertisol in our study had a high SOC sequestration potential. Also, the overall mean SOC sequestration efficiency of the wide range of C inputs via roots, stubble, straw and manure was equally high, with an overall mean efficiency of 16% over the 29-years period. The C sequestration efficiencies of wheat straw and cattle manure are higher than pig manure, indicating that cattle manure or wheat straw application is a recommendable SOC sequestration practice in Vertisols.
